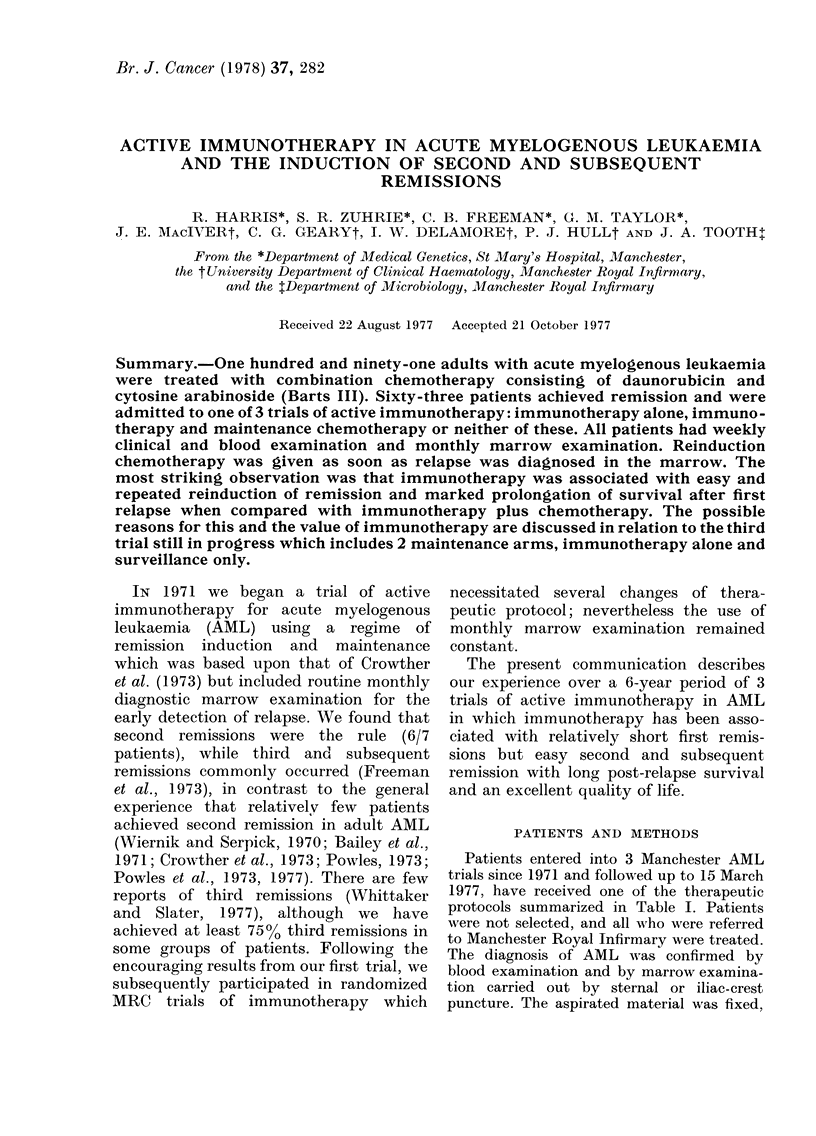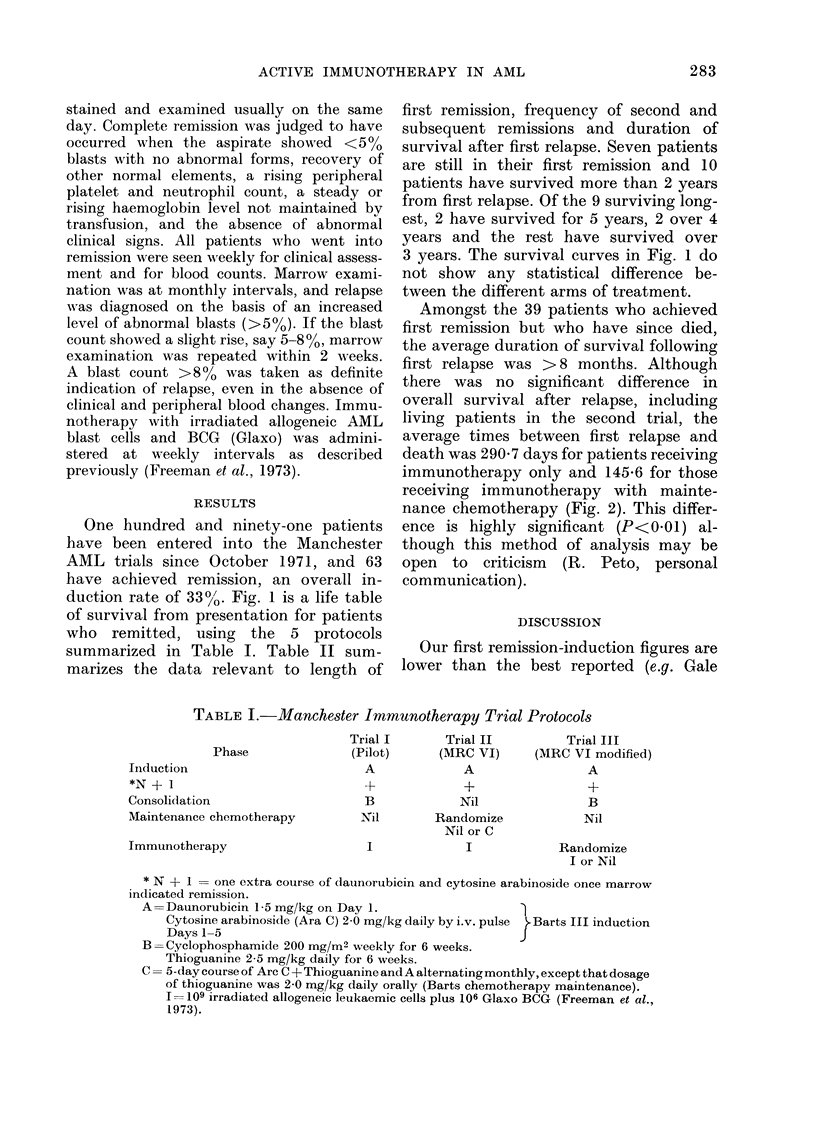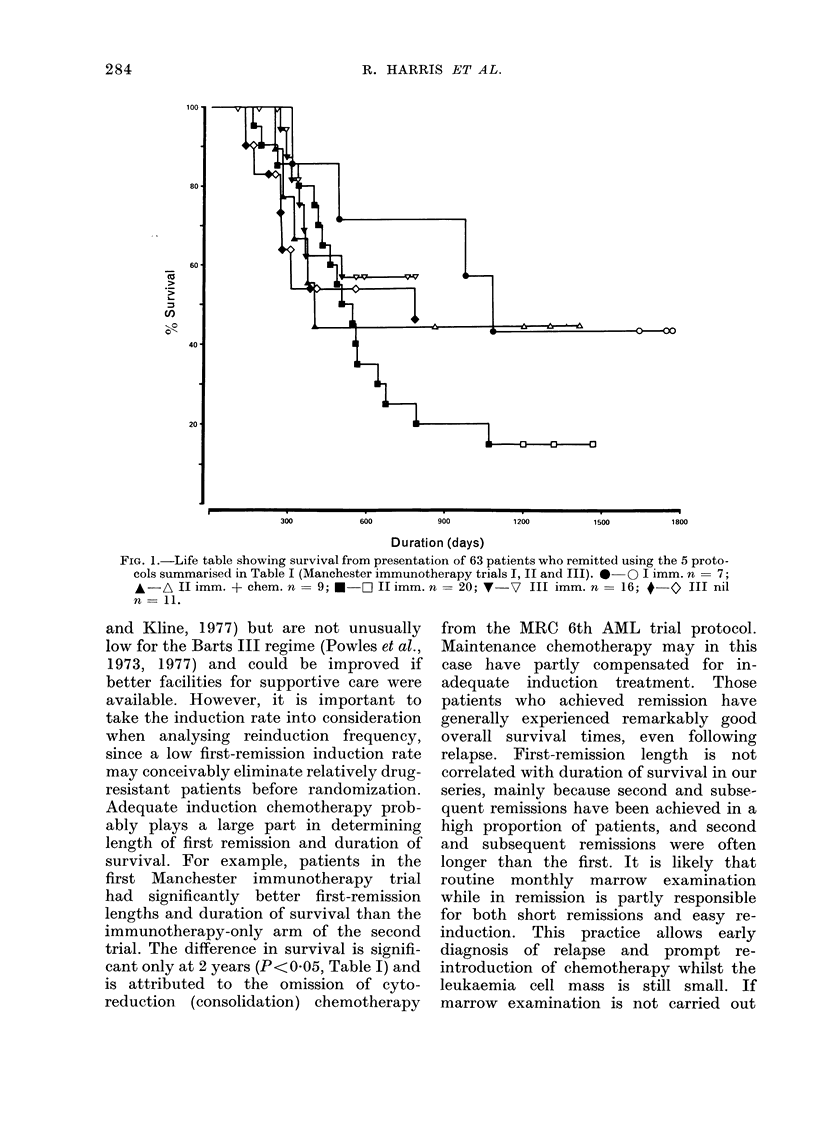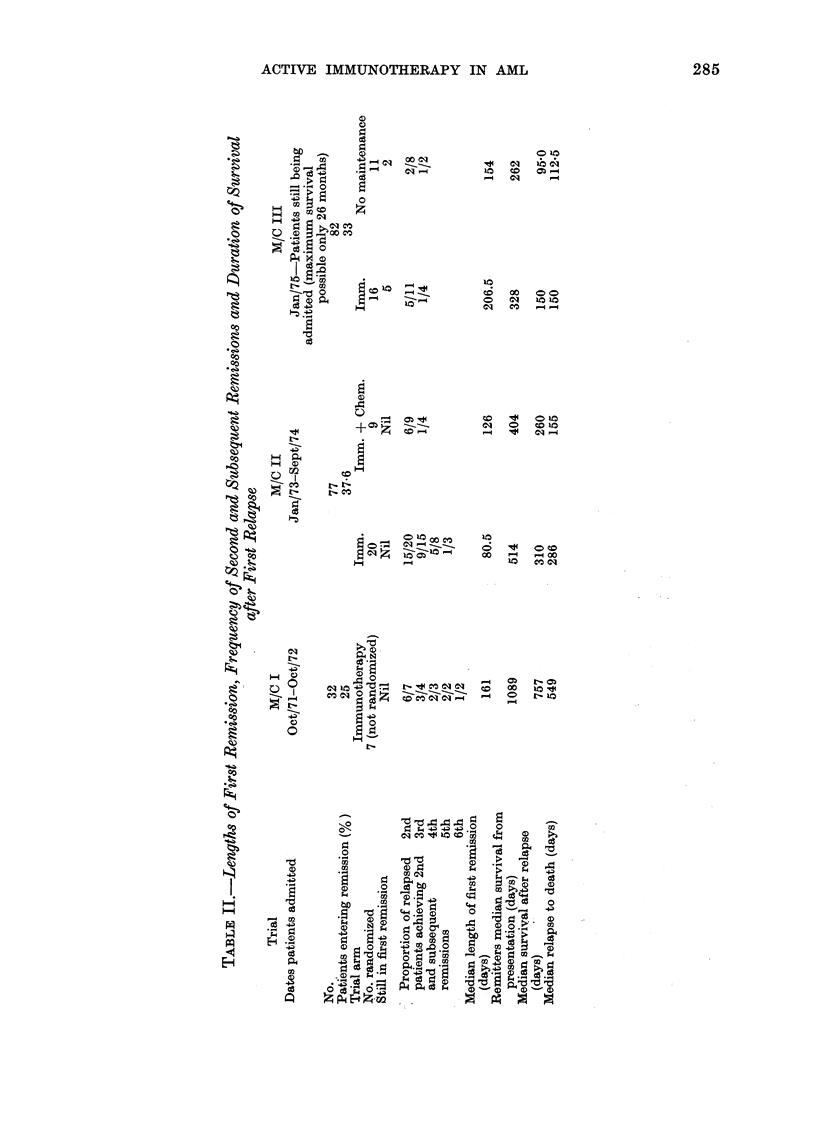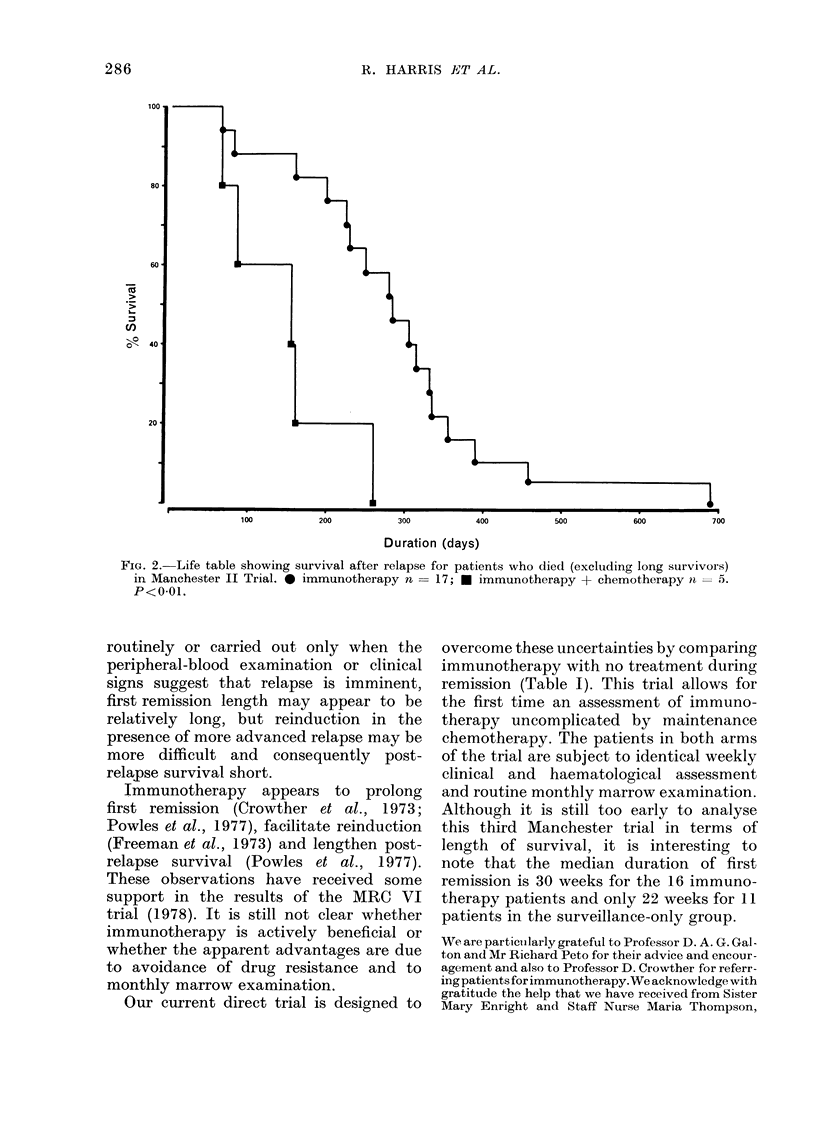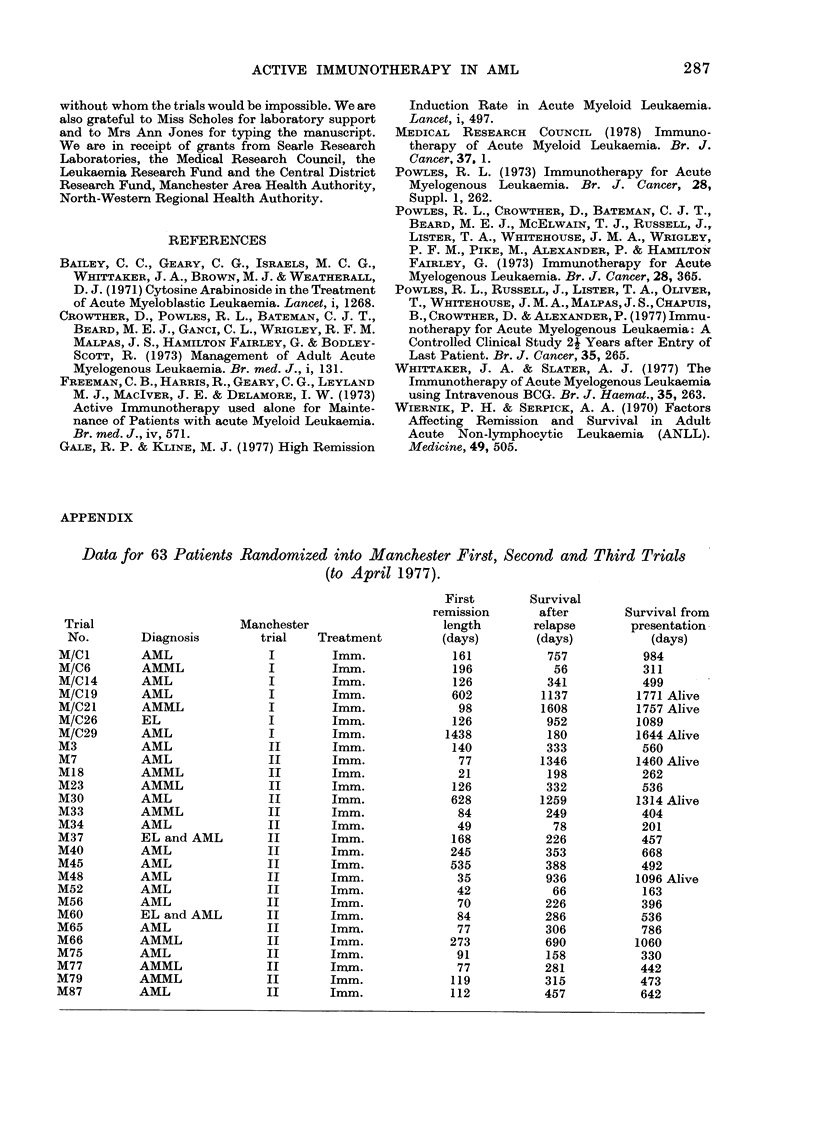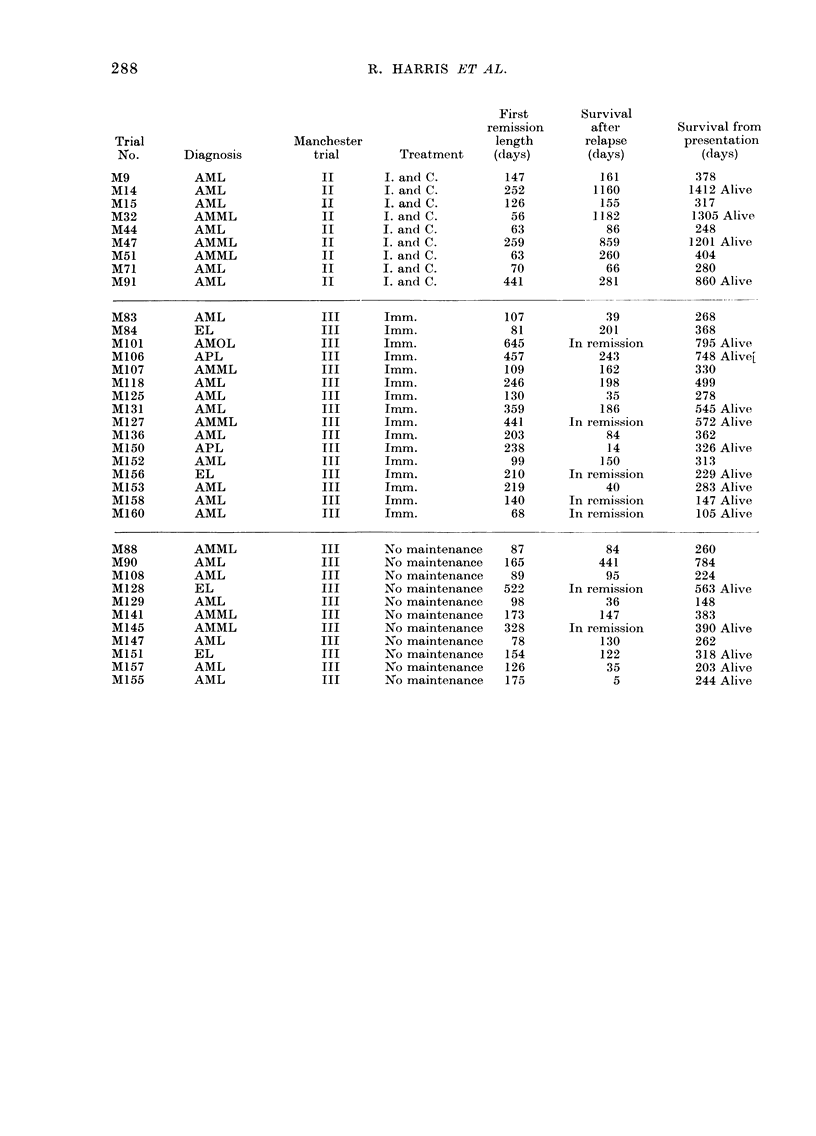# Active immunotherapy in acute myelogenous leukaemia and the induction of second and subsequent remissions.

**DOI:** 10.1038/bjc.1978.37

**Published:** 1978-02

**Authors:** R. Harris, S. R. Zuhrie, C. B. Freeman, G. M. Taylor, J. E. MacIver, C. G. Geary, I. W. Delamore, P. J. Hull, J. A. Tooth

## Abstract

One hundred and ninety-one adults with acute myelogenous leukaemia were treated with combination chemotherapy consisting of daunorubicin and cytosine arabinoside (Barts III). Sixty-three patients achieved remission and were admitted to one of 3 trials of active immunotherapy: immunotherapy alone, immunotherapy and maintenance chemotherapy or neither of these. All patients had weekly clinical and blood examination and monthly marrow examination. Reinduction chemotherapy was given as soon as relapse was diagnosed in the marrow. The most striking observation was that immunotherapy was associated with easy and repeated reinduction of remission and marked prolongation of survival after first relapse when compared with immunotherapy plus chemotherapy. The possible reasons for this and the value of immunotherapy are discussed in relation to the third trial still in progress which includes 2 maintenance arms, immunotherapy alone and surveillance only.


Br. J. Cancer (1978) 37, 282

### ACTIVE IMMUNOTHERAPY IN ACUTE MYELOGENOUS LEUKAEMIA AND THE INDUCTION OF SECOND AND SUBSEQUENT REMISSIONS

R. HARRIS*, S. R. ZUHRIE*, C. B. FREEMAN*, G. M. TAYLOR*, J. E. MacIVER†, C. G. GEARY†, I. W. DELAMORE†, P. J. HULL† AND J. A. TOOTH‡

*From the *Department of Medical Genetics, St Mary's Hospital, Manchester, the †University Department of Clinical Haematology, Manchester Royal Infirmary, and the ‡Department of Microbiology, Manchester Royal Infirmary*

Received 22 August 1977 Accepted 21 October 1977

**Summary.—One hundred and ninety-one adults with acute myelogenous leukaemia were treated with combination chemotherapy consisting of daunorubicin and cytosine arabinoside (Barts III). Sixty-three patients achieved remission and were admitted to one of 3 trials of active immunotherapy: immunotherapy alone, immunotherapy and maintenance chemotherapy or neither of these. All patients had weekly clinical and blood examination and monthly marrow examination. Reinduction chemotherapy was given as soon as relapse was diagnosed in the marrow. The most striking observation was that immunotherapy was associated with easy and repeated reinduction of remission and marked prolongation of survival after first relapse when compared with immunotherapy plus chemotherapy. The possible reasons for this and the value of immunotherapy are discussed in relation to the third trial still in progress which includes 2 maintenance arms, immunotherapy alone and surveillance only.**

In 1971 we began a trial of active immunotherapy for acute myelogenous leukaemia (AML) using a regime of remission induction and maintenance which was based upon that of Crowther *et al.* (1973) but included routine monthly diagnostic marrow examination for the early detection of relapse. We found that second remissions were the rule (6/7 patients), while third and subsequent remissions commonly occurred (Freeman *et al.*, 1973), in contrast to the general experience that relatively few patients achieved second remission in adult AML (Wiernik and Serpick, 1970; Bailey *et al.*, 1971; Crowther *et al.*, 1973; Powles, 1973; Powles *et al.*, 1973, 1977). There are few reports of third remissions (Whittaker and Slater, 1977), although we have achieved at least 75% third remissions in some groups of patients. Following the encouraging results from our first trial, we subsequently participated in randomized MRC trials of immunotherapy which necessitated several changes of therapeutic protocol; nevertheless the use of monthly marrow examination remained constant.

The present communication describes our experience over a 6-year period of 3 trials of active immunotherapy in AML in which immunotherapy has been associated with relatively short first remissions but easy second and subsequent remission with long post-relapse survival and an excellent quality of life.

### PATIENTS AND METHODS

Patients entered into 3 Manchester AML trials since 1971 and followed up to 15 March 1977, have received one of the therapeutic protocols summarized in Table I. Patients were not selected, and all who were referred to Manchester Royal Infirmary were treated. The diagnosis of AML was confirmed by blood examination and by marrow examination carried out by sternal or iliac-crest puncture. The aspirated material was fixed, stained and examined usually on the same day. Complete remission was judged to have occurred when the aspirate showed <5% blasts with no abnormal forms, recovery of other normal elements, a rising peripheral platelet and neutrophil count, a steady or rising haemoglobin level not maintained by transfusion, and the absence of abnormal clinical signs. All patients who went into remission were seen weekly for clinical assessment and for blood counts. Marrow examination was at monthly intervals, and relapse was diagnosed on the basis of an increased level of abnormal blasts (>5%). If the blast count showed a slight rise, say 5–8%, marrow examination was repeated within 2 weeks. A blast count >8% was taken as definite indication of relapse, even in the absence of clinical and peripheral blood changes. Immunotherapy with irradiated allogeneic AML blast cells and BCG (Glaxo) was administered at weekly intervals as described previously (Freeman *et al.*, 1973).

### RESULTS

One hundred and ninety-one patients have been entered into the Manchester AML trials since October 1971, and 63 have achieved remission, an overall induction rate of 33%. Fig. 1 is a life table of survival from presentation for patients who remitted, using the 5 protocols summarized in Table I. Table II summarizes the data relevant to length of first remission, frequency of second and subsequent remissions and duration of survival after first relapse. Seven patients are still in their first remission and 10 patients have survived more than 2 years from first relapse. Of the 9 surviving longest, 2 have survived for 5 years, 2 over 4 years and the rest have survived over 3 years. The survival curves in Fig. 1 do not show any statistical difference between the different arms of treatment.

Amongst the 39 patients who achieved first remission but who have since died, the average duration of survival following first relapse was >8 months. Although there was no significant difference in overall survival after relapse, including living patients in the second trial, the average times between first relapse and death was 290·7 days for patients receiving immunotherapy only and 145·6 for those receiving immunotherapy with maintenance chemotherapy (Fig. 2). This difference is highly significant ($P<0{\cdot}01$) although this method of analysis may be open to criticism (R. Peto, personal communication).

### DISCUSSION

Our first remission-induction figures are lower than the best reported (*e.g.* Gale

TABLE I.—*Manchester Immunotherapy Trial Protocols*

| Phase | Trial I (Pilot) | Trial II (MRC VI) | Trial III (MRC VI modified) |
|---|---|---|---|
| Induction | A | A | A |
| *N + 1 | + | + | + |
| Consolidation | B | Nil | B |
| Maintenance chemotherapy | Nil | Randomize Nil or C | Nil |
| Immunotherapy | I | I | Randomize I or Nil |

* N + 1 = one extra course of daunorubicin and cytosine arabinoside once marrow indicated remission.

A = Daunorubicin 1·5 mg/kg on Day 1. Cytosine arabinoside (Ara C) 2·0 mg/kg daily by i.v. pulse Days 1–5 } Barts III induction

B = Cyclophosphamide 200 mg/m² weekly for 6 weeks. Thioguanine 2·5 mg/kg daily for 6 weeks.

C = 5-day course of Arc C + Thioguanine and A alternating monthly, except that dosage of thioguanine was 2·0 mg/kg daily orally (Barts chemotherapy maintenance).

I = $10^9$ irradiated allogeneic leukaemic cells plus $10^6$ Glaxo BCG (Freeman *et al.*, 1973).

FIG. 1.—Life table showing survival from presentation of 63 patients who remitted using the 5 protocols summarised in Table I (Manchester immunotherapy trials I, II and III). ●—○ I imm. $n = 7$; ▲—△ II imm. + chem. $n = 9$; ■—□ II imm. $n = 20$; ▼—▽ III imm. $n = 16$; ◆—◇ III nil $n = 11$.

and Kline, 1977) but are not unusually low for the Barts III regime (Powles *et al.*, 1973, 1977) and could be improved if better facilities for supportive care were available. However, it is important to take the induction rate into consideration when analysing reinduction frequency, since a low first-remission induction rate may conceivably eliminate relatively drug-resistant patients before randomization. Adequate induction chemotherapy probably plays a large part in determining length of first remission and duration of survival. For example, patients in the first Manchester immunotherapy trial had significantly better first-remission lengths and duration of survival than the immunotherapy-only arm of the second trial. The difference in survival is significant only at 2 years ($P<0{\cdot}05$, Table I) and is attributed to the omission of cytoreduction (consolidation) chemotherapy from the MRC 6th AML trial protocol. Maintenance chemotherapy may in this case have partly compensated for inadequate induction treatment. Those patients who achieved remission have generally experienced remarkably good overall survival times, even following relapse. First-remission length is not correlated with duration of survival in our series, mainly because second and subsequent remissions have been achieved in a high proportion of patients, and second and subsequent remissions were often longer than the first. It is likely that routine monthly marrow examination while in remission is partly responsible for both short remissions and easy reinduction. This practice allows early diagnosis of relapse and prompt reintroduction of chemotherapy whilst the leukaemia cell mass is still small. If marrow examination is not carried out

TABLE II.—*Lengths of First Remission, Frequency of Second and Subsequent Remissions and Duration of Survival after First Relapse*

| Trial | | M/C I | M/C II | | M/C III | |
|---|---|---|---|---|---|---|
| Dates patients admitted | | Oct/71–Oct/72 | Jan/73–Sept/74 | | Jan/75—Patients still being admitted (maximum survival possible only 26 months) | |
| No. | | 32 | 77 | | 82 | |
| Patients entering remission (%) | | 25 | 37·6 | | 33 | |
| Trial arm | | Immunotherapy | Imm. | Imm. + Chem. | Imm. | No maintenance |
| No. randomized | | 7 (not randomized) | 20 | 9 | 16 | 11 |
| Still in first remission | | Nil | Nil | Nil | 5 | 2 |
| Proportion of relapsed patients achieving 2nd and subsequent remissions | 2nd | 6/7 | 15/20 | 6/9 | 5/11 | 2/8 |
| | 3rd | 3/4 | 9/15 | 1/4 | 1/4 | 1/2 |
| | 4th | 2/3 | 5/8 | | | |
| | 5th | 2/2 | 1/3 | | | |
| | 6th | 1/2 | | | | |
| Median length of first remission (days) | | 161 | 80.5 | 126 | 206.5 | 154 |
| Remitters median survival from presentation (days) | | 1089 | 514 | 404 | 328 | 262 |
| Median survival after relapse (days) | | 757 | 310 | 260 | 150 | 95·0 |
| Median relapse to death (days) | | 549 | 286 | 155 | 150 | 112·5 |

FIG. 2.—Life table showing survival after relapse for patients who died (excluding long survivors) in Manchester II Trial. ● immunotherapy $n = 17$; ■ immunotherapy + chemotherapy $n = 5$. $P < 0{\cdot}01$.

routinely or carried out only when the peripheral-blood examination or clinical signs suggest that relapse is imminent, first remission length may appear to be relatively long, but reinduction in the presence of more advanced relapse may be more difficult and consequently post-relapse survival short.

Immunotherapy appears to prolong first remission (Crowther *et al.*, 1973; Powles *et al.*, 1977), facilitate reinduction (Freeman *et al.*, 1973) and lengthen post-relapse survival (Powles *et al.*, 1977). These observations have received some support in the results of the MRC VI trial (1978). It is still not clear whether immunotherapy is actively beneficial or whether the apparent advantages are due to avoidance of drug resistance and to monthly marrow examination.

Our current direct trial is designed to overcome these uncertainties by comparing immunotherapy with no treatment during remission (Table I). This trial allows for the first time an assessment of immunotherapy uncomplicated by maintenance chemotherapy. The patients in both arms of the trial are subject to identical weekly clinical and haematological assessment and routine monthly marrow examination. Although it is still too early to analyse this third Manchester trial in terms of length of survival, it is interesting to note that the median duration of first remission is 30 weeks for the 16 immunotherapy patients and only 22 weeks for 11 patients in the surveillance-only group.

We are particularly grateful to Professor D. A. G. Galton and Mr Richard Peto for their advice and encouragement and also to Professor D. Crowther for referring patients for immunotherapy. We acknowledge with gratitude the help that we have received from Sister Mary Enright and Staff Nurse Maria Thompson, without whom the trials would be impossible. We are also grateful to Miss Scholes for laboratory support and to Mrs Ann Jones for typing the manuscript. We are in receipt of grants from Searle Research Laboratories, the Medical Research Council, the Leukaemia Research Fund and the Central District Research Fund, Manchester Area Health Authority, North-Western Regional Health Authority.

### REFERENCES

BAILEY, C. C., GEARY, C. G., ISRAELS, M. C. G., WHITTAKER, J. A., BROWN, M. J. & WEATHERALL, D. J. (1971) Cytosine Arabinoside in the Treatment of Acute Myeloblastic Leukaemia. *Lancet*, i, 1268.

CROWTHER, D., POWLES, R. L., BATEMAN, C. J. T., BEARD, M. E. J., GANCI, C. L., WRIGLEY, R. F. M. MALPAS, J. S., HAMILTON FAIRLEY, G. & BODLEY-SCOTT, R. (1973) Management of Adult Acute Myelogenous Leukaemia. *Br. med. J.*, i, 131.

FREEMAN, C. B., HARRIS, R., GEARY, C. G., LEYLAND M. J., MACIVER, J. E. & DELAMORE, I. W. (1973) Active Immunotherapy used alone for Maintenance of Patients with acute Myeloid Leukaemia. *Br. med. J.*, iv, 571.

GALE, R. P. & KLINE, M. J. (1977) High Remission Induction Rate in Acute Myeloid Leukaemia. *Lancet*, i, 497.

MEDICAL RESEARCH COUNCIL (1978) Immunotherapy of Acute Myeloid Leukaemia. *Br. J. Cancer*, **37**, 1.

POWLES, R. L. (1973) Immunotherapy for Acute Myelogenous Leukaemia. *Br. J. Cancer*, **28**, Suppl. 1, 262.

POWLES, R. L., CROWTHER, D., BATEMAN, C. J. T., BEARD, M. E. J., MCELWAIN, T. J., RUSSELL, J., LISTER, T. A., WHITEHOUSE, J. M. A., WRIGLEY, P. F. M., PIKE, M., ALEXANDER, P. & HAMILTON FAIRLEY, G. (1973) Immunotherapy for Acute Myelogenous Leukaemia. *Br. J. Cancer*, **28**, 365.

POWLES, R. L., RUSSELL, J., LISTER, T. A., OLIVER, T., WHITEHOUSE, J. M. A., MALPAS, J. S., CHAPUIS, B., CROWTHER, D. & ALEXANDER, P. (1977) Immunotherapy for Acute Myelogenous Leukaemia: A Controlled Clinical Study 2½ Years after Entry of Last Patient. *Br. J. Cancer*, **35**, 265.

WHITTAKER, J. A. & SLATER, A. J. (1977) The Immunotherapy of Acute Myelogenous Leukaemia using Intravenous BCG. *Br. J. Haemat.*, **35**, 263.

WIERNIK, P. H. & SERPICK, A. A. (1970) Factors Affecting Remission and Survival in Adult Acute Non-lymphocytic Leukaemia (ANLL). *Medicine*, **49**, 505.

### APPENDIX

*Data for* 63 *Patients Randomized into Manchester First, Second and Third Trials* (*to April* 1977).

| Trial No. | Diagnosis | Manchester trial | Treatment | First remission length (days) | Survival after relapse (days) | Survival from presentation (days) |
|---|---|---|---|---|---|---|
| M/C1 | AML | I | Imm. | 161 | 757 | 984 |
| M/C6 | AMML | I | Imm. | 196 | 56 | 311 |
| M/C14 | AML | I | Imm. | 126 | 341 | 499 |
| M/C19 | AML | I | Imm. | 602 | 1137 | 1771 Alive |
| M/C21 | AMML | I | Imm. | 98 | 1608 | 1757 Alive |
| M/C26 | EL | I | Imm. | 126 | 952 | 1089 |
| M/C29 | AML | I | Imm. | 1438 | 180 | 1644 Alive |
| M3 | AML | II | Imm. | 140 | 333 | 560 |
| M7 | AML | II | Imm. | 77 | 1346 | 1460 Alive |
| M18 | AMML | II | Imm. | 21 | 198 | 262 |
| M23 | AMML | II | Imm. | 126 | 332 | 536 |
| M30 | AML | II | Imm. | 628 | 1259 | 1314 Alive |
| M33 | AMML | II | Imm. | 84 | 249 | 404 |
| M34 | AML | II | Imm. | 49 | 78 | 201 |
| M37 | EL and AML | II | Imm. | 168 | 226 | 457 |
| M40 | AML | II | Imm. | 245 | 353 | 668 |
| M45 | AML | II | Imm. | 535 | 388 | 492 |
| M48 | AML | II | Imm. | 35 | 936 | 1096 Alive |
| M52 | AML | II | Imm. | 42 | 66 | 163 |
| M56 | AML | II | Imm. | 70 | 226 | 396 |
| M60 | EL and AML | II | Imm. | 84 | 286 | 536 |
| M65 | AML | II | Imm. | 77 | 306 | 786 |
| M66 | AMML | II | Imm. | 273 | 690 | 1060 |
| M75 | AML | II | Imm. | 91 | 158 | 330 |
| M77 | AMML | II | Imm. | 77 | 281 | 442 |
| M79 | AMML | II | Imm. | 119 | 315 | 473 |
| M87 | AML | II | Imm. | 112 | 457 | 642 |

| Trial No. | Diagnosis | Manchester trial | Treatment | First remission length (days) | Survival after relapse (days) | Survival from presentation (days) |
|---|---|---|---|---|---|---|
| M9 | AML | II | I. and C. | 147 | 161 | 378 |
| M14 | AML | II | I. and C. | 252 | 1160 | 1412 Alive |
| M15 | AML | II | I. and C. | 126 | 155 | 317 |
| M32 | AMML | II | I. and C. | 56 | 1182 | 1305 Alive |
| M44 | AML | II | I. and C. | 63 | 86 | 248 |
| M47 | AMML | II | I. and C. | 259 | 859 | 1201 Alive |
| M51 | AMML | II | I. and C. | 63 | 260 | 404 |
| M71 | AML | II | I. and C. | 70 | 66 | 280 |
| M91 | AML | II | I. and C. | 441 | 281 | 860 Alive |
| M83 | AML | III | Imm. | 107 | 39 | 268 |
| M84 | EL | III | Imm. | 81 | 201 | 368 |
| M101 | AMOL | III | Imm. | 645 | In remission | 795 Alive |
| M106 | APL | III | Imm. | 457 | 243 | 748 Alive[ |
| M107 | AMML | III | Imm. | 109 | 162 | 330 |
| M118 | AML | III | Imm. | 246 | 198 | 499 |
| M125 | AML | III | Imm. | 130 | 35 | 278 |
| M131 | AML | III | Imm. | 359 | 186 | 545 Alive |
| M127 | AMML | III | Imm. | 441 | In remission | 572 Alive |
| M136 | AML | III | Imm. | 203 | 84 | 362 |
| M150 | APL | III | Imm. | 238 | 14 | 326 Alive |
| M152 | AML | III | Imm. | 99 | 150 | 313 |
| M156 | EL | III | Imm. | 210 | In remission | 229 Alive |
| M153 | AML | III | Imm. | 219 | 40 | 283 Alive |
| M158 | AML | III | Imm. | 140 | In remission | 147 Alive |
| M160 | AML | III | Imm. | 68 | In remission | 105 Alive |
| M88 | AMML | III | No maintenance | 87 | 84 | 260 |
| M90 | AML | III | No maintenance | 165 | 441 | 784 |
| M108 | AML | III | No maintenance | 89 | 95 | 224 |
| M128 | EL | III | No maintenance | 522 | In remission | 563 Alive |
| M129 | AML | III | No maintenance | 98 | 36 | 148 |
| M141 | AMML | III | No maintenance | 173 | 147 | 383 |
| M145 | AMML | III | No maintenance | 328 | In remission | 390 Alive |
| M147 | AML | III | No maintenance | 78 | 130 | 262 |
| M151 | EL | III | No maintenance | 154 | 122 | 318 Alive |
| M157 | AML | III | No maintenance | 126 | 35 | 203 Alive |
| M155 | AML | III | No maintenance | 175 | 5 | 244 Alive |